# Protection of Pattern Electroretinogram and Retinal Ganglion Cells by Oncostatin M after Optic Nerve Injury

**DOI:** 10.1371/journal.pone.0108524

**Published:** 2014-09-22

**Authors:** Xin Xia, Rong Wen, Tsung-Han Chou, Yiwen Li, Zhengying Wang, Vittorio Porciatti

**Affiliations:** 1 Bascom Palmer Eye Institute, University of Miami, Miller School of Medicine, Miami, Florida, United States of America; 2 Department of Ophthalmology, Shanghai First People’s Hospital, Jiaotong University, Shanghai, China; 3 Shanghai Key Laboratory for Ocular Fundus Diseases, Shanghai, China; Hanson Institute, Australia

## Abstract

Injury to retinal ganglion cell (RGC) axons leads to selective loss of RGCs and vision. Previous studies have shown that exogenous neurotrophic factors promote RGC survival. We investigated the neuroprotective effects of oncostatin M (OSM), a member of the IL-6 family of cytokines, on pattern electroretinogram (PERG) and RGC survival after optic nerve crush (ON-crush) in the mouse. BALB/C mice received ON-crush in the left eyes for either 4-second or 1-second duration (4-s or 1-s). Fluoro-gold retrograde labeling was used to identify RGCs. RGC function was assessed by PERG measurement. OSM or CNTF protein was injected intravitreally immediately after ON-crush. OSM responsive cells were identified by localization of increased STAT3 phosphorylation. Significant higher RGC survival (46% of untreated control) was seen in OSM-treated eyes when assessed 2 weeks after 4-s ON-crush as compared to that (14% of untreated control) of the PBS-treated eyes (P<0.001). In addition, PERG amplitude was significantly higher in eyes treated with OSM or CNTF 1 week after 1-s ON-crush (36% of baseline) as compared with the amplitude of PBS-treated eyes (19% of the baseline, P = 0.003). An increase in STAT3 phosphorylation was localized in Müller layer after OSM treatment, suggesting that Müller cells mediate the effect of OSM. Our results demonstrate that one single injection of either OSM or CNTF after ON-crush improves RGC survival together with their electrophysiological activity. These data provide proof-of-concept for using neurotrophic factors OSM and CNTF for RGC degenerative diseases, including glaucoma and acute optic nerve trauma.

## Introduction

Retinal ganglion cells (RGCs) send neuronal output of the retina to the brain targets through long axons, which form the optic nerve. Injury to RGC axons leads to selective loss of RGCs and vision. The most common disease leading to RGC loss is glaucoma [1], [2], a group of optic neuropathies as the second leading cause of blindness worldwide [3]. Other conditions include ischemia and direct injury to the optic nerve by compression or optic nerve trauma [1].

Axonal transport is crucial for the neuronal survival and function. Impaired axonal transport is now considered as a key factor for the pathogenesis of neurodegenerative disorders [4]. Studies have shown that retrograde axonal transport of target-derived neurotrophic factors, such as BDNF (brain derived neurotrophic factor), is important for RGC survival [5], [6], whereas deprivation of target-derived BDNF by impairment of retrograde axonal transport leading to RGC death [7], [8]. Furthermore, intravitreal administration of neurotrophic factors, including BDNF, NT-4 (neurotrophin-4), and CNTF (ciliary neurotrophic factor), protects RGCs from optic nerve crush induced cell death [9]. These studies support the notion that injury to RGC axons reduces target-derived neurotrophic factors, which in turn leads to RGC death [10]. Promoting RGC survival by neurotrophic factor is a viable strategy for glaucoma and other optic neuropathies [10]–[13]. In fact, a clinical trial of CNTF via CNTF-secreting implants [14] is currently underway (clinicaltrials.gov NCT01408472).

Oncostatin M (OSM) is a member of the IL-6 family of cytokines, including interleukin 6 (IL-6), IL-11, CNTF, leukemia inhibitory factor (LIF), cardiotrophin 1 (CT-1), and cardiotrophin-like cytokine (CLC) [15]–[17]. It was originally isolated from U-937 cells, a histiocytic lymphoma cell line, as a factor that suppresses the growth of tumor cells, hence the name [18]. Like several other members of the family, OSM is a neurotrophic factor. Ablation of OSM gene in mouse results in a significant loss of a subset of nociceptive neurons in the dorsal root ganglia (DRG) and defects in pain sensitivity [16]. In addition, OSM is shown to protect neurons from excitotoxic injury *in* *vitro* and *in* *vivo* [19], [20]. We found recently that OSM protects both rod and cone photoreceptors, and promotes regeneration of cone outer segments in a transgenic rat model of retinal degeneration [21].

The present work examines the potential protective effects of OSM on RGCs and their electrophysiological activity as assessed by PERG. Our results demonstrate clearly that one intravitreal injection of OSM promotes RGC survival and helps to preserve their function after optic nerve crush, similar to CNTF.

## Materials and Methods

### Ethics Statement

All procedures involving animals adhered to Association for Research in Vision and Ophthalmology Statement for the Use of Animals in Ophthalmic and Vision Research and were approved by the Institutional Animal Care and Use Committee of University of Miami, Miller School of Medicine (10–199).

### Animals, RGC labeling, optic nerve crush, and intravitreal injections

BALB/C mice (2 months old) were purchased from Jackson Laboratory (Bar Harbor, ME) and kept on 12∶12 light-dark cycle. Retrograde labeling of RGCs was performed seven days before ON crush. Animals were anesthetized with intraperitoneal ketamine (80 mg/kg) and xylazine (4 mg/kg). The cerebrum was partially aspirated to expose the surface of both superior colliculi (SC). A small piece of gel foam soaked with 3 µL of 2% Fluorogold (FG, Fluorochrome, Denver, CO) [22] was applied over each SC.

ON crush was performed unilaterally while the contralateral eye served as untreated control. After anesthesia with ketamine/xylazine, the skin close to the superior orbital rim was incised, the orbit was opened, and the extraocular muscles were gently separated. The optic nerve was exposed by longitudinal incision of the perineurium [23]. A pair of extra-fine self-closing forceps (RS-5020, tip 0.05×0.01 mm, ROBOZ, MD) was used to crush the optic nerve at 1–2 mm behind the globe for 4 seconds (4-s ON-crush) or for 1 second (1-s ON-crush), without damaging the retinal blood supply.

Intravitreal injections were delivered within 1 minute after ON-crush, through a 33-gauge needle connected to a10-µl microsyringe (Hamilton, Reno, NV), as described previously [24]. The test eyes were injected with human recombinant OSM (3 µg in 2 µl) [21] or CNTF (3 µg in 2 µl) [24]. The control eyes were injected with 2 µl phosphate buffered saline (PBS).

A total of 40 animals were used in the ON-crush experiments, including 12 for 4-s ON-crush, 10 for 1-s ON-crush, 18 for PERG recording. An additional 3 mice were used in the immunohistochemical experiments.

### Quantification of RGCs

**Figure 1:**
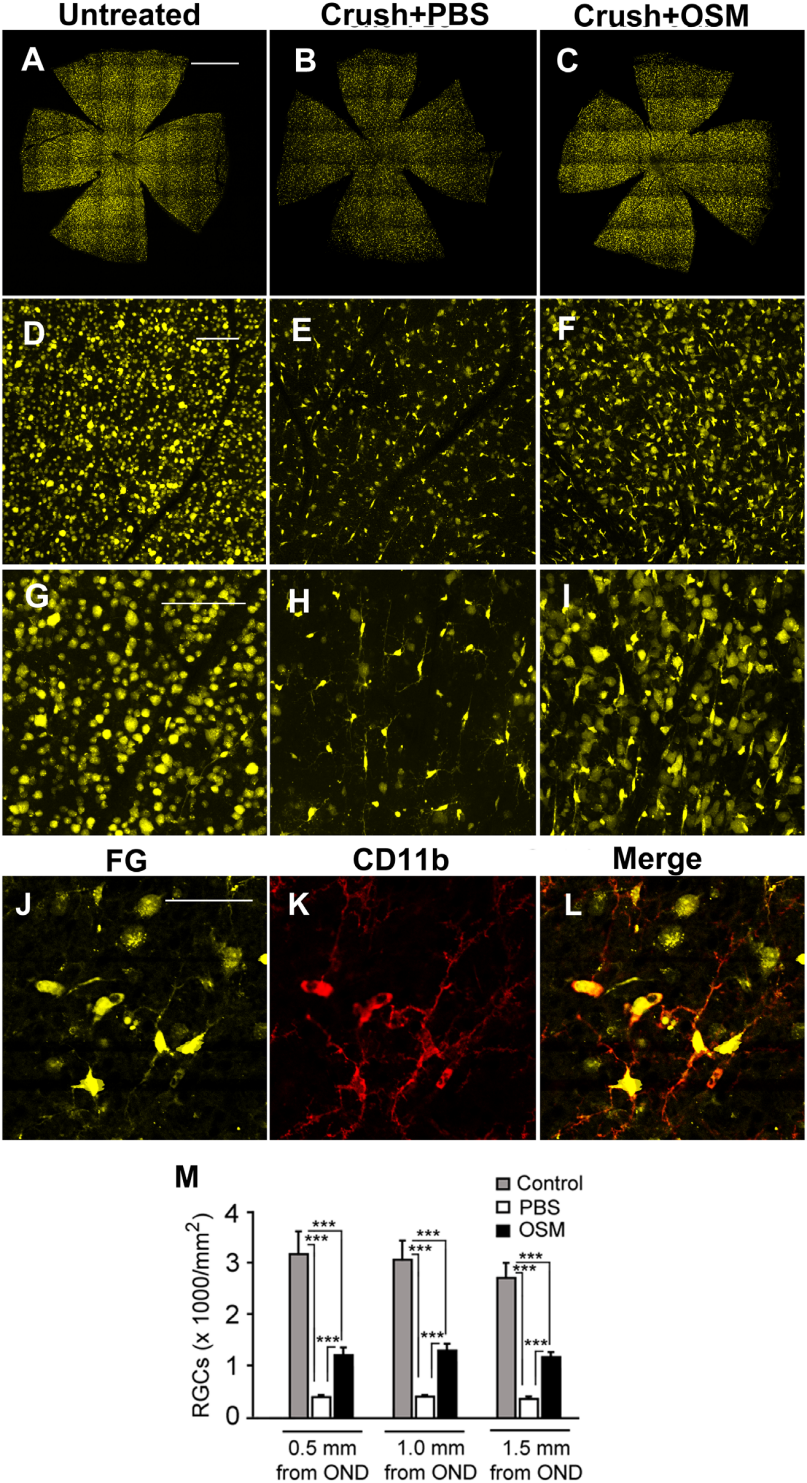
Enhanced survival of RGCs after 4-s ON-crush with OSM treatment. RGCs were retrogradely labeled with Fluorogold (FG). One week after FG labeling, the left eye of an animal received ON-crush (4-s) and treated either with OSM (3 µg in 2 µl, C, F, I) or PBS (2 µl, B, E, H) immediately after ON-crush, while the right eye was untreated and used as control (A, G, D). Two weeks after ON-crush, retinas were harvested for RGC counts. Rod shaped and brightly labeled microglial cells were excluded from RGC counts. Flat-mounted retinas stained with antibodies against CD11b confirmed that those cells were CD11b positive (J, K, L) and thus they were microglias, not RGCs. Quantitative data show a significant loss of RGCs after ON-crush in both PBS- and OSM-treated eyes (Mean+SEM, n = 6, M). However, the remaining RGCs in the OSM-treated eyes were significantly more than in the PBS-treated (J). Scale bars: A–C, 1 mm; D–F, 100 µm; G–I, 100 µm; J–K, 50 µm. Triple asterisks indicate P<0.001.

Retinas were harvested 1 or 2 weeks after ON-crush, fixed in 4% paraformaldehyde, divided into superior, inferior, nasal, and temporal quadrants, flat-mounted onto glass slides, and examined by confocal microscopy (LSM700; Carl Zeiss, Jena, Germany). In each quadrant, FG labeled cells in each eccentricity (0.5, 1 and 1.5 mm from the center of the optic nerve) were counted in 3 sampling fields (0.64×0.64 mm each) in a masked manner. Labeled microglial cells (rod-shaped), (see Fig. 1) [22], [25] were excluded from cell counts.

### PERG recording

PERG recording was performed as described by Porciatti [26]–[28]. Briefly, mice were anesthetized with ketamine/xylazine and gently restrained in an animal holder that allowed unobstructed vision. During recording, animals were kept at a constant body temperature of 37°C using a feedback-controlled heating pad (TCAT-2LV; Physitemp Instruments, Inc. Clifton, NJ). For PERG recording, a silver wire configured to a semicircular loop (2 mm radius) was placed on the cornea. The reference and ground electrodes were placed subcutaneously on the back of the head and the base of the tail, respectively. A visual stimulus of contrast-reversing bars (field area, 69.4°×63.4°; mean luminance, 50 cd/m^2^; spatial frequency, 0.05 cycles/deg; contrast, 98%; temporal frequency, 1 Hz) was aligned with the projection of the pupil at a distance of 15 cm. Signals were amplified (10,000-fold) and bandpass filtered (1–30 Hz). Three consecutive PERG responses to 600 contrast reversals were recorded. The responses (1,800 sweeps) were superimposed to confirm consistency and then averaged. A typical PERG waveform consisted of a main positive wave peaking at about 100 ms followed by a negative wave peaking at about 250 ms, as previously shown [27]. A simple macro written in Sigmaplot language (version 11.2; Systat Software, Inc., San Jose, CA) [27], was used to automatically identify peaks and troughs and measure the peak-to-trough amplitude in a time window 50–300 ms, and to avoid human bias. PERG recordings were interspersed with “noise” recordings obtained with the pattern stimulus occluded. The noise amplitude was also automatically evaluated using the same approach as the PERG. To have a corresponding index of outer retinal function, a light adapted ERG (FERG) was also recorded with undilated pupils in response to strobe flashes of 20 cd/m^2^/s superimposed on a steady background light of 12 cd/m^2^ and presented within a Ganzfeld bowl. Averaged PERG and FERG were automatically analyzed to evaluate the peak-to-trough amplitudes.

### Immunohistochemical analysis

Immunohistochemical analysis of p-STAT3 was performed as previously reported [21]. Mice were perfused 1 hour after intravitreal injection with OSM (3 µg, 2 µL). The retinas were fixed in 4% paraformaldehyde and cryoprotected by 30% sucrose in 0.1 M phosphate buffer. Cryo-sections (16 µm) were stained with antiphospho-STAT3 (phosphor Tyr705) antibodies (Abcam, Cambridge, MA) and visualized using the TSA signal amplification kit (Life Technologies, Grand Island, NY) according to manufacturers’ instructions. Double staining was carried out with either NeuN antibodies (EMD Millipore, Billerica, MA) for RGC, or anti-glutamine synthetase antibodies (EMD Millipore) for Müller cell identification.

To identify microglial cells in the retina after ON-crush, retrograde labeling with FG was performed as described above, followed by 4-s ON-crush 7 days after FG labeling. Retinas were harvested 14 days after ON-crush and stained with antibodies against CD11b (BD Biosciences, San Diego, CA) and Alexa 594 labeled secondary antibodies (Life Technologies, Grand Island, NY).

### Statistical Analysis

Relevant data were graphically displayed and statistically analyzed with Sigmaplot 11.2 (Systat software, Inc. San Jose, CA). Data were presented as the mean±SEM. Statistical analysis was performed by ANOVA. P<0.05 was considered statistically significant.

## Results

### Enhanced RGC survival by OSM after optic nerve crush

The mouse ON-crush model was used to investigate whether OSM could enhance the survival of RGCs. RGCs were first retrogradely labeled with Fluoro-gold in both eyes. One week later, in the treated group, the left eye of an animal received 4-second ON-crush and intravitreal injection of OSM (3 µg in 2 µl) immediately after ON-crush, whereas the right eye received neither ON-crush nor injection. In the control group, the left eyes received 4-second ON-crush and intravitreal injection of PBS (2 µl). Two weeks after ON-crush, retinas were harvested for RGC counts.

Figure 1 shows representative fluorescence photomicrographs. Many RGCs with typical oval-shaped somata were seen in eyes without ON-crush (Fig. 1 A, D, G). RGC density was much lower in the eyes received ON-crush+PBS than that of untreated eyes (Fig. 1, B, E, H). In addition, many microglial cells (smaller size, rod-shaped) [22], [25] were present (Fig. 1, B, E, H). However, significantly more RGCs were found in eyes treated with ON-crush+OSM compared to the ON-crush+PBS treated eyes (Fig. 1 C, F, I). In RGC quantification, labeled microglial cells were excluded. The microglial cells appeared as rod shaped and labeled with FG brighter than RGCs. Staining with CD11b, a microglia specific marker [29], confirmed that they were microglias (Fig. 1, J, K, L). Quantitative data (Fig. 1M, n = 6 for each group) show the mean RGC densities in eyes without ON-crush decreased with increasing eccentricity, consistent with previous studies [30]. In eyes that received ON-crush+PBS, the mean RGC densities were much lower at all retinal eccentricities compared to eyes without ON-crush (13.7% of normal at 0.5 mm, 14.1% at 1.0 mm, and 14.6% at 1.5 mm). In eyes treated with ON-crush+OSM, however, RGC densities were significantly higher than eyes treated with ON-crush+PBS (42.0% of normal at 0.5 mm, 47.2% at 1.0 mm, and 48.1% at 1.5 mm; two-way ANOVA: effect of treatment, P<0.001). Thus, OSM treatment resulted in significant better RGC survival two weeks after ON-crush.

### Protection of PERG by OSM and CNTF after optic nerve crush

**Figure 2:**
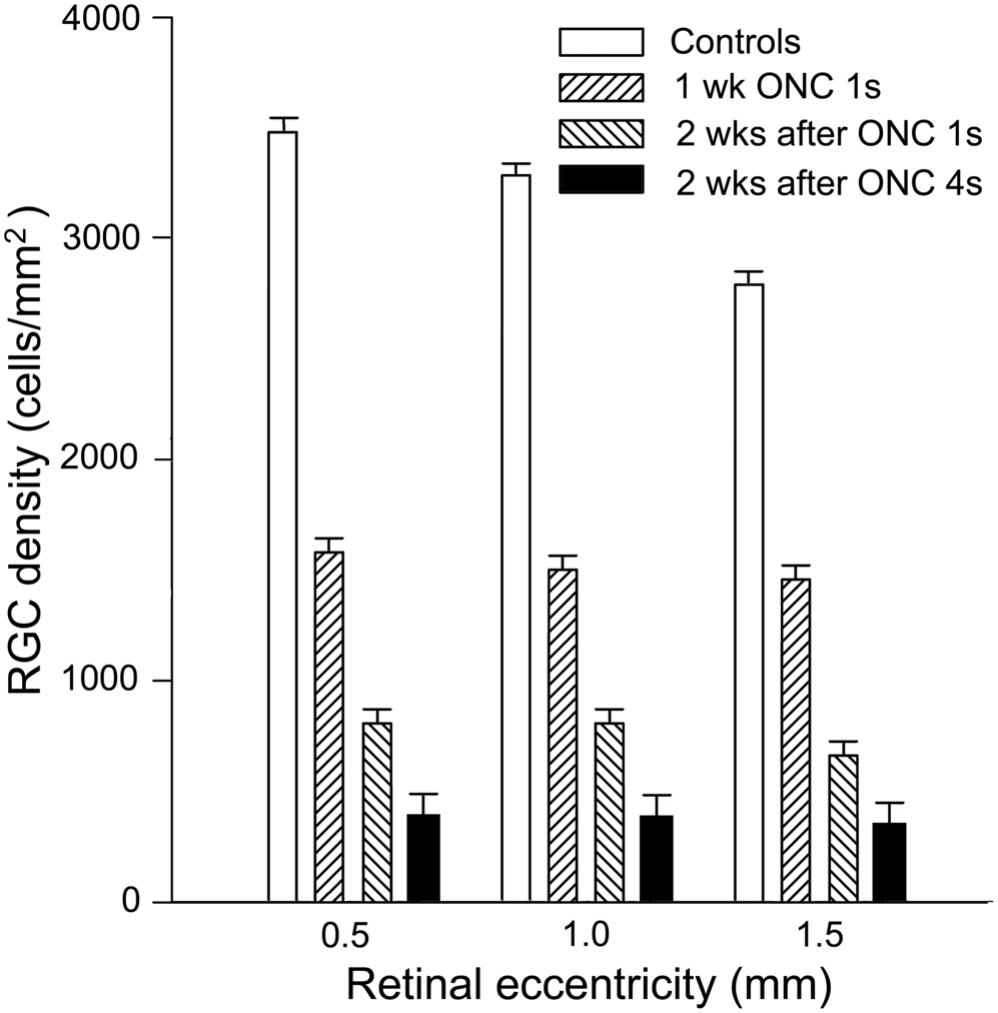
RGC loss after 1-s ON-crush. RGCs were retrogradely labeled with Fluorogold and ON-crush (1-s) was performed one week later. At least 48% of RGCs survived 1 week after ON-crush at all eccentricities, and at least 24% survived 2 weeks after ON-crush (mean+SEM, n = 5). The difference between 1-s and 4-s ON-crush on RGC survival at 2 weeks was significant (P<0.01, 2 way ANOVA). In comparison, only 14% RGCs left 2 weeks after 4-s ON-crush (n = 6).

The mouse PERG is reported to be very sensitive to optic nerve injury [31], [32]. Intraorbital ON-crush (5 second crush) rapidly and irreversibly abolishes the PERG in mice [33]. In our pilot experiments, we confirmed that the PERG was virtually flat after a 4-s ON-crush (not shown). In order to have a residual dynamic range of the PERG signal after ON-crush to assess the protective effects of OSM, we attempted a milder (1-s duration) ON-crush. Indeed, two weeks after ON-crush, more RGCs survived with 1-s ONC compared to 4-s ON-crush (P<0.001, two-way ANOVA) (Fig. 2). In addition, close to half of RGCs survived one week after 1-s ON-crush, with the mean RGC survival of 46%, 46%, 52% at increasing eccentricities of 0.5 mm, 1.0 mm, and 1.5 mm from optic nerve head. Altogether, these results indicate less RGC death and thus better survival after 1-s ON-crush than 4-s ON-crush (Fig. 2).

**Figure 3:**
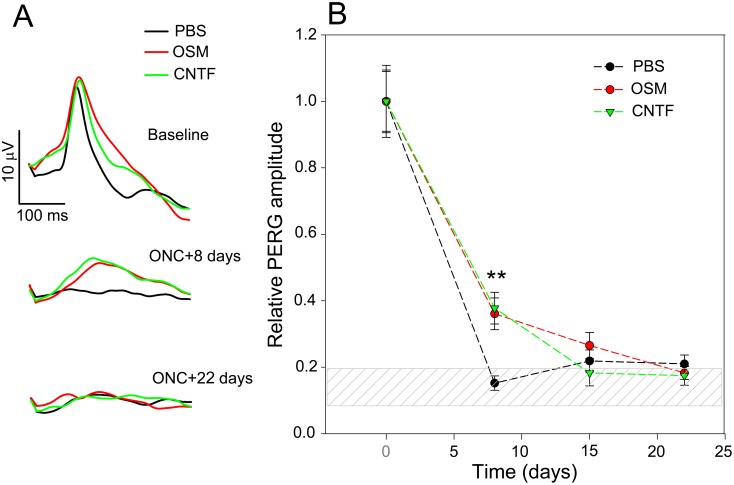
Effect of OSM or CNTF treatment on PERG after ON-crush. OSM (3 µg in 2 µl) or CNTF (3 µg in 2 µl) was delivered intravitreally immediately after 1-s ON-crush. Eyes of control animals received PBS (2 µl). PERG was recorded before ON-crush as baseline and at 8, 15, 22 days after ON-crush. A: grand-average PERG waveforms recorded in PBS-, OSM-, and CNTF-treated eyes before and at different times after ON-crush. B: mean PERG amplitude as a function of time in PBS-, OSM-, and CNTF-treated groups. Eight days after ON-crush, the PERG amplitude was in the noise range (hatched area) in the PBS-treated group. However, in both OSM- and CNTF-treated groups, the PERG amplitudes were significantly higher (P = 0.003) than that of the PBS-treated group. By day 15 and 22, the PERG amplitudes were at or close to the noise range in all groups. Error bars represent the SEM (n = 6).

To establish a baseline, PERG and FERG (photopic flash ERG) were recorded from all eyes of the three groups (n = 6 each) before ON-crush. For the two test groups, OSM (3 µg in 2 µl) or CNTF (3 µg in 2 µl) was injected intravitreally into the left eyes immediately after ON-crush. PBS (2 µl) was injected into left eyes of the control group in the same manner. Follow-up PERGs and FERGs were recorded at 8, 15, 22 days after ON-crush. Figure 3A shows grand-average PERG waveforms for each group. Baseline PERG waveforms and amplitudes in BALB/C mice ranged between 15 and 22 µV, consistent with previous findings for C57BL/6J mice [27]. Eight days after ON-crush, the PERG was flat in PBS-treated eyes, but significantly higher PERG amplitude was recorded in eyes treated with either OSM or CNTF, although lower than the baseline levels. All groups had flat PERG 22 days after ON-crush (Fig. 3A). To compare longitudinal changes among different groups, PERG amplitudes of individual mice were normalized to the mean baseline amplitude of each group (Fig. 3B). Eight days after ON-crush in PBS-treated eyes, PERG amplitude decreased sharply to 15% of the baseline level. In both OSM- and CNTF-treated eyes, however, the PERG amplitudes were above the noise range and significantly higher (P = 0.003) than that of the PBS-treated eyes (Fig. 3B). By day 15, the PERG amplitudes of OSM- and CNTF-treated eyes decreased to the levels close to the noise range, not statistically different from PBS-treated eyes. The PERG amplitudes of all groups were in the noise range 22 days after ON-crush and no significant difference was found among the three groups.

For FERG, no significant changes were observed over time after ON-crush, consistent with previously reported data [33], nor after intraocular injections of OSM or CNTF (data not shown).

### Localization of OSM-induced STAT3 phosphorylation

**Figure 4:**
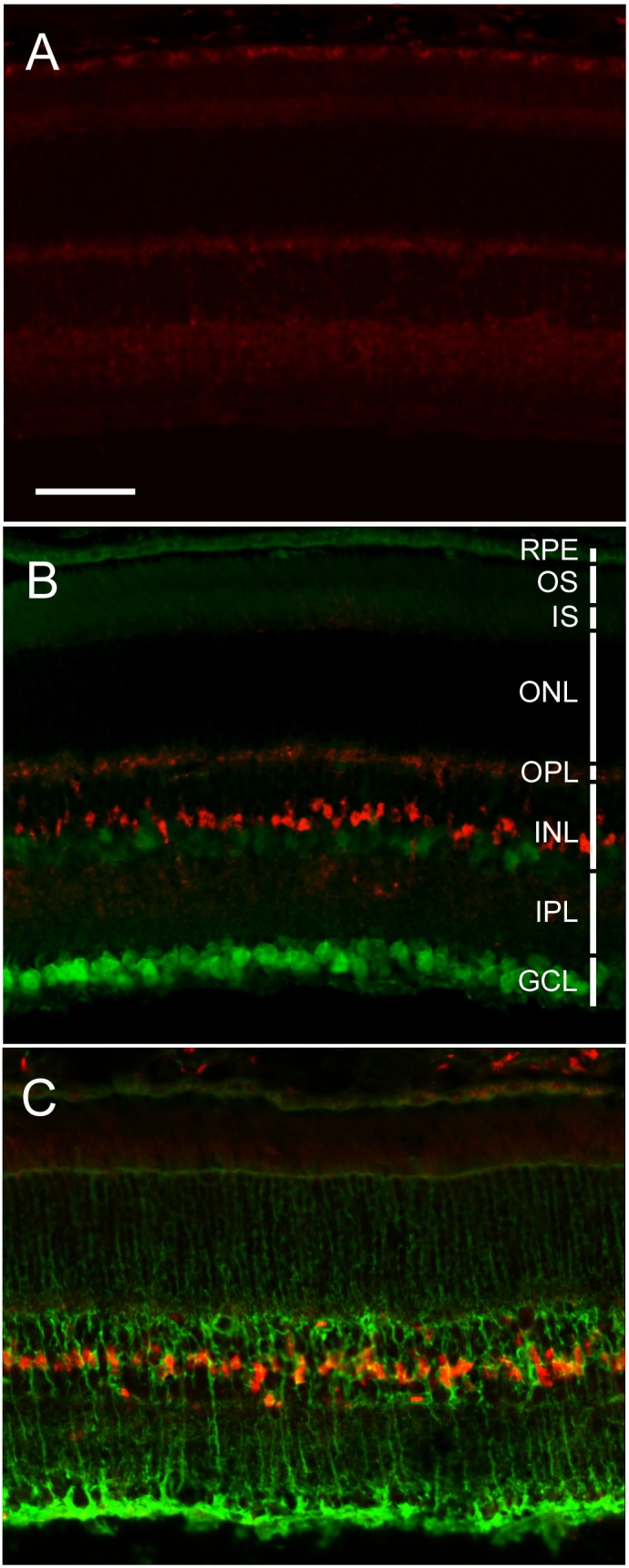
Increase in STAT3 phosphorylation in Müller cells after OSM treatment. Eyes were intravitreally injected with either PBS (2 µl) or OSM (3 µg in 2 µl), and retinas were harvested 1 hour later. Cryosections (16 µm) were stained for phosphorylated STAT3 (pSTAT3). Although no specific pSTAT3 staining was detected in the PBS treated control retina (A), a substantial increase in pSTAT3 immunoreactivity was seen in the OSM-treated retinas (B, C). The increased pSTAT3 is not co-localized with the RGC marker NeuN (B), but well co-localized with the Müller cell marker glutamine synthetase (C). RPE, retinal pigment epithelium; OS: photoreceptor outer segments; IS: photoreceptor inner segments; ONL: outer nuclear layer; OPL: outer plexiform layer; INL: inner nuclear layer; IPL: inner plexiform layer; GCL: ganglion cell layer. Scale bar: 50 µm.

Like CNTF, the biological activity of OSM is mediated through activation of STAT3 and thus OSM responsive cells can be identified by localizing OSM-induced STAT3 phosphorylation, as shown previously [21]. To localize OSM-induced STAT3 phosphorylation, mice were intravitreally injected with OSM (3 µg in 2 µl) in the left eyes and PBS (2 µl) in the right eyes. Retinas were harvested 1 hour later. Phosphorylated STAT3 was detected in the OSM-treated retina in a group of cells in the inner nuclear layer (INL) (Figure 4B, C), whereas no specific staining of phosphor-STAT3 was visible in the PBS-treated retina (Fig. 4A). Double staining using antibodies against neuron-specific nuclear protein (NeuN), an RGC marker [34], [35], showed no co-localization of phosphor-STAT3 immunoreactivity with NeuN signals (Fig. 4B). On the other hand, the immunostaining signals of glutamine synthetase, a Müller cell marker [24], co-localized well with those of phosphor-STAT3 (Fig. 4C). These results are consistent with our previous observations that OSM induces STAT3 activation in Müller cells [21].

## Discussion

The present work has demonstrated the neuroprotective effects of OSM and CNTF on RGC survival and RGC function in a mouse model of intraorbital optic nerve crush. ON-crush in rodents causes marked loss of RGCs in two weeks [36], [37] and is a widely used model of nerve injury to investigate mechanisms of RGC death, neuroprotection, and RGC axon regeneration [9], [38]–[40]. However, functional aspects of RGC cell injury after ON-crush have not been extensively studied. Our results provide strong evidence that OSM, as well as CNTF, not only improves RGC survival, but also partially preserves their function. These data support the hypothesis that injury to RGC axons reduces target-derived neurotrophic factors, which in turn leads to RGC death [10]. Furthermore, these results provide a proof-of-concept for potential treatments for RGC degenerative disorders with OSM or CNTF, as well as their potential use as an emergency treatment for traumatic injury to the optic nerve to save vision.

Our results showed clearly that longer duration of ON-crush causes more damage to the optic nerve, as the 1-s ON-crush resulted in a significantly higher RGC survival than the 4-s ON-crush. Still, the PERG signal was reduced to the noise range by 8 days after 1-s ON-crush in PBS-treated eyes, indicating that the PERG is particularly vulnerable to ON-crush injury. The presence of a recordable PERG signal in both OSM- and CNTF-treated eyes 8 days after ON-crush indicates that both factors are effective in preserving of the electrophysiological responsiveness of RGCs, in addition to promoting their survival. Loss of PERG signal after ON-crush may mean that a population of RGCs is missing or dead, that RGCs are surviving but dysfunctional, or a combination of both. Our results cannot establish quantitative relationships between these two conditions.

Although the preservation of PERG signals with OSM or CNTF treatment was significant 8 days after ON-crush, it was no longer observed by day 15 and 22. The limited duration of OSM and CNTF treatment was likely due to the short half-lives of the injected proteins. Studies in rat show that the maximal effect of CNTF on photoreceptors is detectable 6 days after a single intravitreal injection of a large amount of CNTF protein (10 µg), and it is fully reversed in 3 weeks [24]. Intravenously injected CNTF in rat undergoes a biphasic decrease with an initial half-life of 2.9 minutes, followed by a slower half-life of 4 hours [41]. It is not clear how long the intravitreal half-life of injected OSM was in our experiments. However, it is unlikely to be very different from that of CNTF, judging from the similar temporal pattern of their effects. These results emphasize the importance of sustained delivery of neurotrophic factors for potential clinical application.

The magnitude of PERG preservation by OSM treatment was similar to that of CNTF, a neurotrophic factor that has been extensively studied for its effects on retinal cells [42]. The neuroprotective effects of OSM have been demonstrated in models of excitotoxic injury [19], [20]. Our previous finding that OSM protects photoreceptors is consistent with OSM being a neurotrophic cytokine [21]. As a member of the IL-6 family of cytokines, OSM has significant homology of protein sequence and structure with other family members [15], [43]. In mouse, human OSM activates the heterodimer of LIF receptor β (LIFRβ) and gp130 [44], like CNTF [45], [46]. Our data showed that OSM induces the STAT3 pathway in Müller cells, but not in RGCs, suggesting that the neuroprotecive effect of OSM on RGCs is mediated by Müller cells.

In summary, the present work clearly shows that OSM improves RGC survival and preserves their electrophysiological function after ON-crush. These data provide preclinical data for potential therapeutic approaches using OSM and CNTF for optic nerve diseases.
